# Comparison of risk variables associated with the metabolic syndrome in pre- and postmenopausal Bengalee women

**Published:** 2008

**Authors:** Arnab Ghosh

**Affiliations:** Department of Anthropology, Palli Charcha Kendra, Visva Bharati University, Santiniketan, West Bengal, India

## Abstract

**Background:**

Clustering of risk variables associated with the metabolic syndrome (MS) begins before the onset of menopause. However, studies of the factors underlying this clustering have focused on only postmenopausal women.

**Aim:**

The present community-based, cross-sectional investigation was aimed at identifying the principal components of risk variables associated with the metabolic syndrome in pre- and postmenopausal Bengalee women.

**Methods:**

A total of 200 (100 premenopausal women; mean age 5 40.2 ± 6.5 years and 100 postmenopausal women; mean age 5 55.4 ± 5.2 years) healthy adult (30 years and older) Bengalee women took part in the study. Obesity measures, metabolic profiles and blood pressures were taken. Principal components factor analysis (PCFA) was used to identify the principal components of the MS.

**Results:**

There were significant differences between the two groups for obesity measures, metabolic profiles and blood pressure, even after adjusting for age. PCFA revealed three uncorrelated factors with a 67.1% explanation in the premenopausal women. Four factors, with overlapping between the first three factors, and a 73% explanation were evident for the postmenopausal women.

**Results:**

Since more than one factor was identified, more than one physiological mechanism could have accounted for clustering of the risk variables associated with the MS and this would warrant early intervention, well before the menopause.

## Summary

Coronary heart disease (CHD) remains the major cause of mortality in women after menopause,^1^ which, regardless of the age of onset, is associated with a marked increase in CHD risk.^2^,^3^ An early natural menopause poses an excess risk of CHD, and a decrease in risk in women who take oestrogen replacement further demonstrates that the excess risk of CHD in women is a consequence of oestrogen deficiency.^3^,^4^ However, how these effects are mediated remains unclear. One hypothesis is that endogenous oestrogen deficiency leads to an adverse lipid profile, which in turn results in an enhanced risk of CHD in postmenopausal women.^5^

There are twin epidemics occurring in postmenopausal women, one being CHD and the other the metabolic syndrome (MS), between which there appears to be a connection.^6^-^8^ Metabolic risk factors such as elevated plasma triglyceride levels, decreased HDL cholesterol levels and glucose abnormalities have been suggested to portend a greater CHD risk in postmenopausal women,^6^,^9^ with the inflammatory markers, C reactive protein (CRP) and others appearing to modulate risk at all levels of the MS.^10^

In India, cardiovascular disease (CVD) has been projected to be the largest cause of death and disability by 2020, with about 29.8 million people estimated to have CHD in 2003 and the prevalence of stroke thought to be 203 per 100 000 population among people older than 20 years.^11^ The prevalence of CHD (one of the best-known components of CVD) is known to be high in people of Indian origin.^12^-^17^ Indians of both genders who have settled in the United States have a four-fold higher prevalence of CHD than Caucasians or Americans, and a six-fold higher hospitalisation rate than that of Chinese Americans.^13^,^14^,^18^ Moreover, CHD occurs at least a decade earlier in these Indians than in Europeans or Americans.

The reason for the increased susceptibility of Indians to CHD is yet to be completely understood. However, studies have suggested that the clustering of more risk variables of the MS in Indians than in other populations could be one possible reason why there is increasing incidence of CHD in people of Indian origin. This includes glucose intolerance, central obesity, hypertriglyceridaemia, dyslipidaemia and insulin resistance.^16^,^17^,^19^,^20^ A report by WHO/IASO/IOTF,^21^ exclusively prepared for the Asia−Pacific region, stated that in Asian populations, mortality and morbidity from CVD is occurring with a lower body mass index (BMI). Therefore they tend to accumulate intra-abdominal fat (central obesity) without developing generalised obesity.^12^

As far as Asian Indians are concerned, very few studies have been undertaken so far to identify the components of the metabolic syndrome. Moreover, there is evidence that Indian women may be comparatively worse off than men with regard to many of the risk factors for CHD.^22^,^23^ Comparison of pre- and postmenopausal women in this regard would therefore be useful to better comprehend the conditions.

To the author’s knowledge, no study has been undertaken to compare pre- and postmenopausal Asian Indian women (e.g. Bengalee women) for commonly considered risk factors of the MS. No attempt has been made either to identify the cluster of components that are commonly used as MS risk factors before and after the occurrence of menopause in this population. With these in mind, pre- and postmenopausal Bengalee women were compared for risk factors often seen in the MS, and principal component factor analysis (PCFA) was also undertaken to identify the components of risk variables (clusters) associated with the MS in this population.

## Materials and methods

The study was initiated in the first week of December 1999 and was continued to the last week of December 2005. A total of 200 (premenopausal: 100 and postmenopausal: 100) healthy adult (30 years and older) Bengalee women were subjects in the investigation. This sample size was sufficient to test all the research hypotheses at the 5% level of significance with a power of 80% (β 5 0.80). A random sampling procedure using a local voters’ registration list was followed to select the subjects. Primary information including name, address and age of randomly selected individuals was collected from the same registration list. Prior to actual commencement of the study, written information was communicated to selected individuals and an appointment was requested at their respective homes.

Out of 300 (premenopausal:postmenopausal 5 1:1) randomly selected individuals, a total of 100 subjects were excluded due to chronic illness such as hypothyroidism (40%), or their inability to participate in the study because of time constraints (60%). Pregnant women, those on hormone replacement therapy (HRT) as well as women with known illness such as ischaemic heart disease (IHD), diabetes and hypertension were not included in the study.

Women were defined as postmenopausal if they had reported their last menses to be at least 12 months previously, and premenopausal if they had had an unchanged and regular menstrual pattern during the last five years, without typical climacteric complaints. A schedule on health and menopausal status written in the local language was used.^24^

Demographic profiles including name, date of birth, educational levels and gross annual family income were obtained from participants. Subjects’ age to the nearest month was ascertained subsequently from date of birth. All subjects were resident in Calcutta and its suburbs. Informed consent was obtained from participants prior to the actual commencement of the study. The institutional ethics committee approved the study.

Anthropometric measures such as height (to the nearest to 0.1 cm), weight (to the nearest to 0.5 kg) and circumference of waist (WC) and hip were recorded using standard procedures.^25^ Waist circumference was taken as the narrowest part of the torso as seen from the anterior aspect. Body mass index (BMI) and waist−hip ratio (WHR) were subsequently computed.

A fasting blood sample was collected from each subject for the determination of metabolic variables. All subjects maintained an overnight fast (≥ 10 hours) prior to blood collection. Within two hours of collection, plasma was separated by centrifugation at 1 000 3 g for 20 minutes at room temperature. Estimation of total cholesterol (TC), fasting triglycerides (FTG) and fasting plasma glucose (FPG) was carried out on separated plasma using a Technicon RA-XT auto-analyser (Technicon Instrument Corporation, NY, USA). High-density lipoprotein cholesterol (HDL) was measured after standing the plasma overnight in a refrigerator, and then precipitation of non-high-density lipoproteins was done with a manganese−heparin substrate. Values of low-density lipoprotein cholesterol (LDL-C) were estimated using the following standard equation:^26^

$LDL=TC-HDL+\frac{FTG}{5}$

All metabolic variables were measured in mg/dl (mg%) and then converted into mmol/l using standard conversion units.

Left arm systolic (SBP) and diastolic (DBP) blood pressure was taken from each participant with the help of an Omron M1 digital electronic blood pressure/pulse monitor (Omron Corporation, Tokyo, Japan). Two blood pressure measurements were taken and averaged for analysis. A third measurement was taken when the difference between the two measurements was ≥ 5 mmHg, and a subsequent mean was calculated. A five-minute relaxation period between measurements was maintained for all participants. The working condition of the instrument was checked periodically using a mercury sphygmomanometer and stethoscope (auscultator procedure).

## Statistical analyses

The distribution of variables was checked for normality. Log_10_ transformation was undertaken to normalise the distribution of positively skewed variables (WHR, TC, FTG, LDL and FPG). Descriptive statistics such as mean and standard deviation (SD) of anthropometric, metabolic and blood pressure measures were undertaken separately for the two groups. Group (pre- vs postmenopausal women) differences for the variables were tested using analysis of co-variance (ANCOVA), adjusting for age.

Principal component factor analysis (PCFA) is one approach that groups quantitatively measured variables into clusters known as factors, based on correlations between variables.^27^ For example, if there is a single underlying cause of clustering of risk variables of the MS, then factor analysis should produce only one major factor or component. Identification of components of the MS is therefore necessary to better comprehend the aetiology of CHD.

Factor analysis was undertaken in three steps: computation of a correlation matrix for all variables included; factor extraction; and orthogonal rotation to make factors readily interpretable. Factors were extracted by PCFA, in which linear combinations of variables were formed, with the first principal component accounting for the largest amount of variance in the sample. Varimax rotation, an orthogonal rotation in which the factors are assumed to act independently (maximum likelihood), was used in the study.

Factor loadings, which were equivalent to Pearson’s correlation coefficient between each variable and the factor were used to interpret each factor. Factor loadings with an absolute value of 0.4 or greater were used to interpret the rotated factor patterns, as suggested elsewhere.^16^,^20^,^27^-^30^ A factor loading of 0.4 or greater was used to interpret the final rotated factor pattern in the present study. Extracted factors (extraction by PCFA) were such that each explained at least as much (eigenvalue ≥ 1) or nearly as much variance as any one observed variable (eigenvalue 5 1).

All statistical analyses were performed using the SPSS version 10 package. A *p*-value of < 0.05 (two-tailed) was considered statistically significant.

## Results

Mean and standard deviations (SD) of anthropometric, metabolic and blood pressure measures are presented in Table 1. The mean age of pre- and postmenopausal women was 40.2 ± 6.5 and 55.4 ± 5.2 years, respectively. ANCOVA (adjusted for age) revealed significant group (pre- vs postmenopausal women) differences for WC (*p* < 0.01), BMI (*p* < 0.05), WHR (*p* < 0.001), TC (*p* < 0.05), FTG (*p* < 0.01), HDL (*p* < 0.05), LDL (*p* < 0.001), FPG (*p* < 0.001), SBP (*p* < 0.001) and DBP (*p* < 0.01).

**Table 1:** Characteristics Of The Study Population

Variable	*Premenopausal women (n 5 100)*		*Postmenopausal women (n 5 100)*	
	*Mean*	*SD*	*Mean*	*SD*
Waist circumference (cm)**	84.8	3.2	88.6	2.8
Body mass index (kg/m^2^)*	22.4	2.6	23.2	2.2
Waist−hip ratio***	0.88	0.04	0.93	0.07
Total cholesterol (mg/dl)*	205.4	16.4	218.0	18.6
Triglycerides (mg/dl)**	108.2	14.4	122.4	16.3
High-density lipoprotein cholesterol (mg/dl)*	45.2	4.6	43.6	4.2
Low-density lipoprotein cholesterol (mg/dl) ***	127.4	20.2	136.2	22.2
Fasting plasma glucose (mg/dl)***	102.4	12.6	123.0	14.3
Systolic blood pressure (mmHg)***	134.5	18.0	145.0	17.4
Diastolic blood pressure (mmHg)**	82.4	13.4	87.6	14.6

Premenopausal women, mean age 5 40.2 ± 6.5 years; postmenopausal women, mean age 5 55.4 ± 5.2 years. Significant group (age adjusted) differences at *p < 0.05; **p < 0.01; ***p < 0.001.

**Fig. 1.**
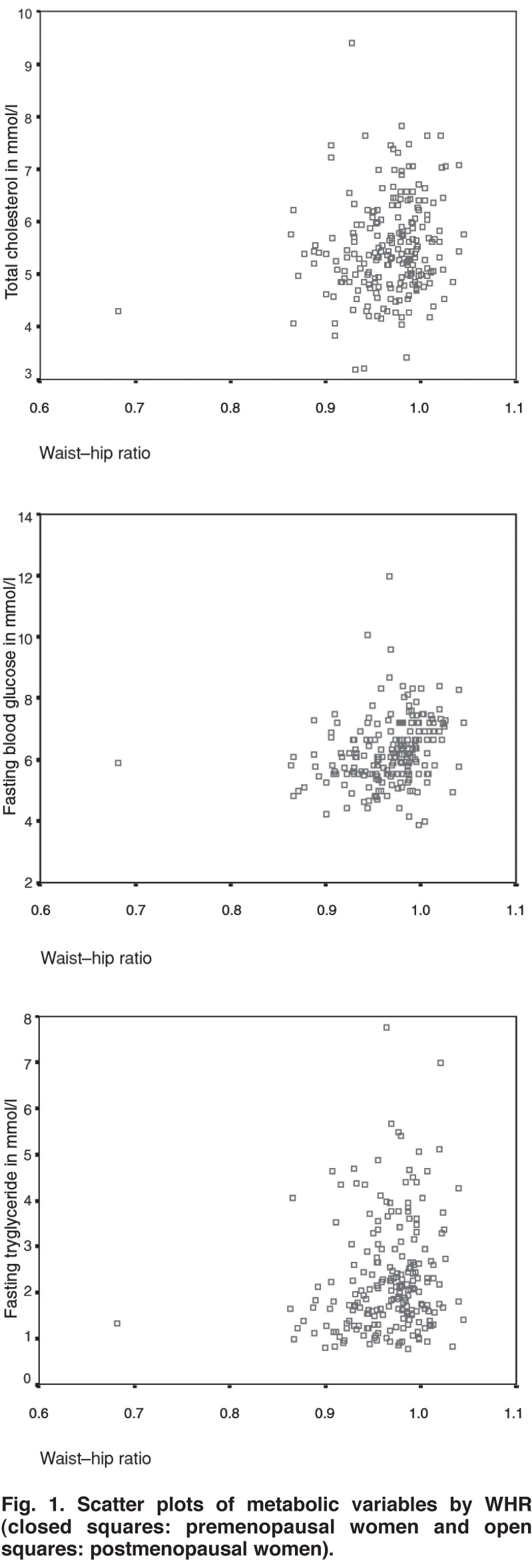
Scatter plots of metabolic variables by WHR (closed squares: premenopausal women and open squares: postmenopausal women).

The intercorrelation matrix of obesity measures, lipids, lipoproteins, blood glucose and blood pressure variables is presented in Table 2. BMI had no significant association with central obesity measures (WC, WHR), metabolic (TC, FTG, HDL, LDL and FPG) or blood pressure variables. On the other hand, central obesity measures had a significant positive association (except for HDL) with metabolic and blood pressure variables. Scatter plots of the metabolic variables by WHR are presented in Fig. 1. It was observed that distribution of TC, FTG and FBG by WHR was almost similar for pre- and postmenopausal women.

**Table 2:** Intercorrelation Matrix

	*WC*	*BMI*	*WHR*	*TC*	*FTG*	*HDL*	*LDL*	*FPG*	*SBP*	*DBP*
WC	–	0.11	0.90	0.60	0.64	−0.28	0.26	0.51	0.46	0.48
BMI		–	0.12	0.12	0.13	−0.13	0.12	0.14	0.13	0.15
WHR*			–	0.67	0.66	−0.44	0.48	0.64	0.52	0.46
TC*				–	0.65	−0.68	0.75	0.60	0.32	0.37
FTG*					–	−0.28	0.36	0.47	0.52	0.55
HDL						–	−0.45	0.42	−0.36	−0.38
LDL*							–	0.44	0.35	0.34
FPG*								–	0.50	0.52
SBP									–	0.76
DBP										–

WC 5 waist circumference; BMI 5 body mass index; WHR 5 waisthip ratio; TC 5 total cholesterol; FTG 5 fasting triglycerides; HDL 5 high-density lipoprotein cholesterol; LDL 5 low-density lipoprotein cholesterol; FPG 5 fasting plasma glucose; SBP 5 systolic blood pressure; DBP 5 diastolic blood pressure. *Log_10_ transformed values were used. Significant at: 5% level when coefficient was > 0.18; 1% level when coefficient was > 0.26; 0.1% level when coefficient was > 0.32.

The factor-loading pattern of components identified is presented in Table 3. Only variables with loading ≥ 0.4 were considered for explanations. PCFA with varimax rotation revealed three uncorrelated factors for premenopausal women that cumulatively explained 67.1% of the observed variance of the metabolic syndrome. Four factors, with factor 2 overlapping with both factors 1 and 3, were observed for postmenopausal women, and in turn cumulatively explained 73.0% of the total variation of the MS. The three factors for premenopausal women were central obesity (WC and WHR, factor 1), lipids−blood glucose (TC, FTG, HDL, LDL and FPG, factor 2), and blood pressure (SBP and DBP, factor 3).

**Table 3:** Factor Loading By Principal Component Analysis With Varimax Rotation

	*Premenopausal women (n 5 100)*			*Postmenopausal women (n 5 100)*			
	*Factors*			*Factors*			
*Variables*	*F-1*	*F-2*	*F-3*	*F-1*	*F-2*	*F-3*	*F-4*
WC	0.820*	0.324	0.321	0.804*	0.721*	0.326	0.312
BMI	0.210	0.189	0.215	0.301	0.289	0.311	0.287
WHR^b^	0.776*	0.218	0.356	0.779*	0.712*	0.356	0.253
TC^b^	0.313	0.778*	0.311	0.314	0.278	0.771*	0.216
FTG_b_	0.314	0.887*	0.345	0.288	0.322	0.659*	0.287
HDL	0.341	0.882*	0.312	0.232	0.268	0.688*	0.314
LDL	0.289	0.886*	0.331	0.322	0.256	0.658*	0.243
FPG_b_	0.288	0.860*	0.312	0.311	0.713*	0.747*	0.288
SBP	0.228	0.332	0.885*	0.256	0.288	0.362	0.867*
DBP	0.231	0.312	0.861*	0.315	0.248	0.333	0.831*
Variance explained (%)	26.3	24.4	16.4	23.5	22.0	13.0	14.5
Cumulative variance (%)	26.3	50.7	67.1	23.5	45.5	58.5	73.0

WC 5 waist circumference; BMI 5 body mass index; WHR 5 waist−hip ratio; TC 5 total cholesterol; TG 5 triglycerides; HDL = highdensity lipoprotein cholesterol; LDL 5 low-density lipoprotein cholesterol; FPG 5 fasting plasma glucose; SBP 5 systolic blood pressure; DBP 5 diastolic blood pressure. ^b^Log_10_ transformed values were used. *Loading with absolute value ≥ 0.4.

On the other hand, four factors identified in postmenopausal women were central obesity (WC and WHR, factor 1), central obesity−plasma glucose (WC, WHR and FPG, factor 2), lipids−blood glucose (TC, FTG, HDL, LDL and FPG, factor 3), and blood pressure (SBP and DBP, factor 4). The loading of individual risk variables varied from 0.65 to 0.88. Age was not incorporated as an independent or explanatory variable during factor extraction owing to significant group differences (pre- vs postmenopausal women).

## Discussion

This investigation aimed to compare risk variables of the metabolic syndrome in pre- and postmenopausal women, and also to identify components (using PCFA) of the metabolic syndrome in pre- and postmenopausal Bengalee women.

Comparison (adjusted for age) of pre- and postmenopausal women revealed significant differences in lipid metabolism, plasma glucose and blood pressure between subjects who differed primarily in their menopausal state and therefore in endogenous sex hormone production. In a study in 2004, hyperandrogenism was associated with the metabolic syndrome and this increased the risk of cardiovascular disease in premenopausal women.^31^ However, the relation between endogenous androgens and the MS in postmenopausal women is complex and the role of androgen in lipid−obesity homeostasis in postmenopausal women requires further study.

Although the HDL concentration was significantly higher in premenopausal compared to postmenopausal women, low HDL concentrations (< 40 mg%) were absent in both groups. This could be attributed to the frequent consumption of fish as staple food by both groups.

PCFA identified three uncorrelated factors for premenopausal women that cumulatively explained 67.1% of the observed variance of the metabolic syndrome. Four factors, with overlapping between the first three factors, were evident for postmenopausal women that eventually (cumulatively) explained 73% of the total variation of the metabolic syndrome. A constellation of central obesity measures and plasma glucose as second factor for only postmenopausal women indicated that elevated plasma glucose along with increasing central obesity increased the risk for the metabolic syndrome during menopause. PCFA had revealed an almost similar cluster of factors for both groups. This indicates that the premenopausal women were no less vulnerable for risk factors of the metabolic syndrome than the postmenopausal women.

In a study on postmenopausal women with CHD, the definition for impaired fasting glucose (proposed in 2003) was not associated with increased risk for new CHD, stroke or transient ischaemic attack (TIA) or congestive heart failure (CHF).^1^ In another study on French women aged 45 to 65 years, it was observed that postmenopausal women had significantly higher plasma levels of total cholesterol, LDL and triglycerides, and lower levels of HDL than perimenopausal women, independent of age, BMI and years since menopause.^32^ Postmenopausal Bengalee women also had significantly higher body fat levels, plasma lipids (except HDL), blood glucose and blood pressure than their premenopausal counterparts in the present investigation. A number of studies has also shown that premenopausal women had greater HDL concentrations than postmenopausal women.^22^,^32^-^34^

These studies have highlighted that preventive interventions to reduce dyslipidaemia and control other coronary risk factors can reduce CHD mortality and morbidity after the onset of menopause. Furthermore, overlapping of central obesity and glucose components indicated that central obesity (WHR) rather than overall fat (BMI) is a stronger predictor of the metabolic syndrome in postmenopausal women. Moreover, overlapping of central obesity and glucose components was also evident in overweight premenopausal women.^34^,^35^ There is also an indication that Asian Indian women may be comparatively worse off than men in many aspects of risk for CHD.^22^,^23^

It is noteworthy, however, to mention that the results of factor analysis are limited by differences in ethnicity, gender and age composition of the study samples, the number of risk variables included, sample size, and the cutoff point of loadings set by the investigators. A study to associate the expression of genes and menopause would be interesting to better comprehend the gene−hormone interaction in the MS.

This study’s major limitation was that it was performed on a relatively small sample size and therefore it is not representative of the Asian Indian population. Owing to considerable ethnic and cultural heterogeneity in the Asian Indian population, it is necessary to study other ethnic groups to see if the observed trends also exist among them. Results obtained from such studies could be utilised to prevent increasing incidence of the MS in women, especially after the onset of menopause. To the author’s knowledge, no study in this respect has been attempted on the Indian subcontinent. The lack of assessment of sex steroid concentrations further limits the interpretation of any group (pre- vs postmenopausal) differences in clustering of risk factors of the metabolic syndrome in this study.

## Conclusion

Significant differences in lipid metabolism, plasma glucose and blood pressure were found in subjects who differed primarily in their menopausal state and therefore in their endogenous sex hormone production. The higher concentration of plasma lipids, lipoproteins (except HDL) and plasma glucose, and higher blood pressure associated with the postmenopausal state seems one possible reason to explain their contribution to increased risk of the metabolic syndrome after menopause, and this warrants intervention as early as the onset of menopause.
